# What influences a patient’s decision to use custom-made orthopaedic shoes?

**DOI:** 10.1186/1471-2474-13-92

**Published:** 2012-06-08

**Authors:** Jaap J van Netten, Pieter U Dijkstra, Jan H B Geertzen, Klaas Postema

**Affiliations:** 1 Department of Rehabilitation Medicine, Center for Rehabilitation, University Medical Center Groningen, University of Groningen, Groningen, the Netherlands; 2 Department of Surgery, Hospital Group Twente, Almelo, the Netherlands; 3 Department of Oral and Maxillofacial Surgery, University Medical Center Groningen, University of Groningen, Groningen, the Netherlands

## Abstract

**Background:**

Despite potential benefits, some patients decide not to use their custom-made orthopaedic shoes (OS). Factors are known in the domains ‘usability’, ‘communication and service’, and ‘opinion of others’ that influence a patient’s decision to use OS. However, the interplay between these factors has never been investigated. The aim of this study was to explore the interplay between factors concerning OS, and the influences thereof on a patient’s decision to use OS.

**Methods:**

A mixed-methods design was used, combining qualitative and quantitative data by means of sequential data analysis and triangulation. Priority was given to the qualitative part. Qualitative data was gathered with a semi-structured interview covering the three domains. Data was analysed using the framework approach. Quantitative data concerned the interplay between factors and determining a rank-order for the importance of factors of ‘usability’.

**Results:**

A patient’s decision to use OS was influenced by various factors indicated as being important and by acceptance of their OS. Factors of ‘usability’ were more important than factors of ‘communication’; the ‘opinion of others’ was of limited importance. An improvement of walking was indicated as the most important factor of ‘usability’. The importance of other factors (cosmetic appearance and ease of use) was determined by reaching a compromise between these factors and an improvement of walking.

**Conclusions:**

A patient’s decision to use OS is influenced by various factors indicated as being important and by acceptance of their OS. An improvement of walking is the most important factor of ‘usability’, the importance of other factors (cosmetic appearance and ease of use) is determined by reaching compromises between these factors and an improvement of walking. Communication is essential to gain insight in a patient’s acceptance and in the compromises they are willing to reach. This makes communication the key for clinicians to influence a patient’s decision to use OS.

## Background

Custom-made orthopaedic shoes (OS) are prescribed to enhance mobility of a patient, for example by reducing pain in feet or ankles or preventing ulceration [1]. OS are prescribed to patients with a wide range of pathologies, such as diabetes, rheumatoid disorders, and degenerative foot disorders [1]. OS are prescribed frequently in England and Wales (about 3800 pairs per million inhabitants at a total cost of 40 million pounds sterling [2]), and in the Netherlands (about 3100 pairs per million inhabitants at a total cost of 60 million Euros [3]). A basic prerequisite for OS to enhance mobility is their actual use. Despite the potential benefits, some patients decide not to use their OS [4-6].

Some insight into the reasons for non-use of OS is available from quantitative and qualitative studies [4-11]. From these, three domains emerge. Each domain highlights a number of factors that may influence a patient’s decision to use OS: i) ‘usability of OS’, ii) ‘communication and service of clinicians’ (clinicians are the medical specialist and the orthopaedic shoe technician), and iii) the ‘opinion of others’ [4-11]. Examples of factors of ‘usability’ that are known to be positively associated with use of OS are: an increase in mobility; a reduction of pain; increased comfort; and a positive opinion of the patient on their cosmetic appearance [4-8,11]. Examples of factors of ‘communication and service’ that are known to be positively associated with use of OS are: the patient’s feeling that the clinicians listen to them; patient’s involvement in the prescription process; and a good relationship between the patient and their clinician(s) [5,9,10,12].

How these factors might connect, or the way in which one factor might influence another has never been investigated. It is therefore not clear whether one factor is considered to be more important than another for any individual patient. However, research into other assistive technologies (AT) has found that taking into account what is of most importance for an individual patient is crucial for increasing the likelihood that they decide to use their AT [13-15]. Therefore, investigation of the interplay between factors of importance in use of OS for an individual patient might lead to answers over why they decide to use their OS or not.

There is currently limited evidence available on the interplay between these factors. Therefore, a mixed-methods design with priority to the qualitative part to obtain a richness of data useful for the generation of ideas is required [16,17]. The aim of this study was to explore the interplay between factors of use of OS, and the influences these might have on a patient’s decision to use their OS.

## Methods

This study was approved by the Medical Ethics Committee of the University Medical Center Groningen.

### Participants

Inclusion criteria were: i) received first-ever pair of OS between two and four months before the interview; ii) 16 years of age or older; iii) able to speak Dutch. In total, 23 participants were included in this study (Table 1).

**Table 1:** Participant characteristics (n = 23)

Age (years)	mean ± SD	67 ± 10
Pathology	Diabetes	22% (5)
	Rheumatoid disorder	22% (5)
	Degenerative foot disorder	26% (6)
	Oedema	13% (3)
	Other	17% (4)
Use of OS	Always	83% (19)
	Never	17% (4)

Note: Values are % (n); *OS* = custom-made orthopaedic shoes.

Two orthopaedic shoe companies agreed to recruit participants, independent from the investigators. The company representative sent an invitation letter explaining the research to all clients who met the inclusion criteria. An informed consent form was included, which participants could return to the investigators in a pre-stamped envelope, if they were willing to participate. After receiving informed consent, participants were contacted by the investigators to arrange an appointment for the interview.

### Study design

A mixed-methods design was used, combining qualitative and quantitative data by means of sequential data analysis and triangulation [16]. Qualitative data was gathered in a semi-structured interview. This interview was conducted with each participant by one investigator (JvN), who received interview training before undertaking this research. The interviews were carried out in each participant’s home. The interviews were audiotape recorded, and transcribed verbatim afterwards.

The interview consisted of three sections, based around each domain of use of OS, in the following order: ‘usability of OS’; ‘communication and service of clinicians’; ‘opinion of others’. Each section began with the question: what is important for you? Subsequently, for each factor they indicated was important, questions were asked on the participant’s experiences with that factor, the interplay between it and other factors, and the influence it had on their decision to use OS.

After the qualitative part, all factors indicated by the patient as being important were listed. After 21 interviews, no new factors emerged during the qualitative part, indicating that enough patients were included to draw valid conclusions [17]. Two more interviews were conducted to confirm this, after which no more appointments for further interviews were made.

**Figure 1:**
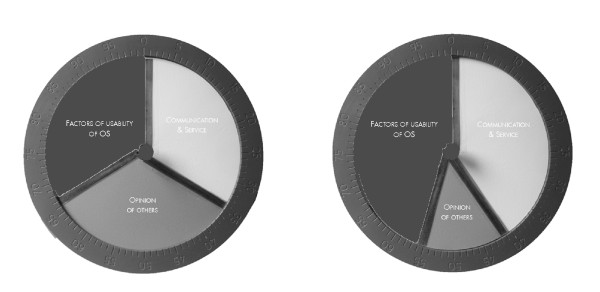
**Disc used to measure the relative importance of the three domains of the interview **[18]**.** Note: Two examples are shown. Left: this patient gave all three domains equal relative importance. Right: this patient gave the domains ‘factors of usability of OS’ and ‘communication and service’ almost equal relative importance, whereas the domain ‘opinion of others’ was considered to be less important. The score was measured from the second disc with the 100-point scale, which was placed around the circumference after the patient indicated to be satisfied with the result.

Quantitative data was gathered after the interview. The interplay between factors in the three domains of the interview was measured using a technique based on the Schedule for Evaluation of Individual Quality of Life, Direct Weighting Procedure [18]. We used a tool, consisting of three stacked, centrally mounted, interlocking discs, each in a different colour, representing all factors of ‘usability, ‘communication and service’, ‘opinion of others’ respectively [18] (Figure 1). Essentially, it is a dynamic pie chart where the relative size represented the importance attached to factors in that domain. Participants rotated the discs until they were satisfied with the position and weightings assigned to each disc. A second disc was placed around the circumference of the measurement tool, to calculate a score on a 100-point scale. Scores can vary from 0 to 100; the sum of the three discs is always 100. Finally, a rank-order was asked for all factors of ‘usability’ the patient had indicated as being important.

### Data analysis

Qualitative data was analysed following five phases of the framework approach [17]. During these phases, raw data is condensed, rearranged, and finally, to enable analysis and interpretation, mapped into a thematic framework that is developed specifically for each individual study [17].

#### Phase 1: familiarisation

After verbatim transcription of the interviews by one investigator (JvN), a meeting was held with all four investigators to discuss key ideas and recurrent themes.

#### Phase 2: identifying a thematic framework

Based on the discussion in phase 1, a thematic framework was identified. All factors that had been described by the patients were grouped into themes within each of the three domains. A unique numerical code was given to all factors as part of the coding system.

#### Phase 3: indexing

Two investigators (JvN and KP) coded one interview to test the coding system. The coding system was found to be satisfactory, and seven interviews were coded by two investigators (JvN and KP) subsequently. Similarities and differences in coding were discussed. Almost all coding was identical, and agreement was reached over minor differences found. The remaining 16 interviews were coded by one investigator (JvN).

#### Phase 4: charting

The coded data was rearranged in a matrix according to the thematic framework developed in phase 2.

#### Phase 5: mapping and interpretation

The matrix, with all coded data, was analysed separately by two investigators (JvN and JG). Similarities and differences in their findings were discussed. Both had reached similar interpretations and conclusions, with agreement by discussion over some minor differences. As an extra step, two independent physicians with experience in the field of OS also analysed the matrix of the coded data. They too obtained similar interpretations and conclusions.

All quotes presented in the results section were translated from Dutch into English by a native English speaker. This was done in cooperation with one investigator (JvN), to ensure that the translations captured the original content in context of the interview.

Quantitative data was analysed by calculating the mean and standard deviation for the relative importance of factors in the three domains (indicated from the dynamic pie chart). Additionally, the frequency of each factor of ‘usability’ indicated as important was recorded. Finally, the findings from both the qualitative and quantitative parts were compared and triangulated for final conclusions [16].

## Results

### Qualitative results

#### Interplay between all factors

Factors of ‘usability’ were consistently described as most important in comparison to factors of ‘communication and service’ and ‘opinion of others’. Factors of ‘usability’ determine whether the main goal of OS can be met. Therefore it always has an influence on a patient’s decision to use their OS.

"Female pt B: “Yes, it works, it really works. And as soon as I take them off, I’ve got a problem.”"

Factors of ‘communication and service’ were considered as less important in relation to the decision to use OS. However, good communication increased satisfaction, which had an indirect but positive effect on a patient’s decision to use their OS. This was further exemplified when patients were asked what they would do if confronted with a problem with their OS. Those who were satisfied with communication indicated they would go back and request changes, continuing to use their OS thereafter, whereas those who were dissatisfied with communication indicated they would not go back and would cease using their OS.

"Female pt A: “I would continue to discuss the problem with them, until the OS fit perfectly.”"

"Male pt H: “You get dissatisfied, and you think, whatever, forget it. And then you don’t bother to return.”"

The ‘opinion of others’ was deemed as being of little importance, with no one indicating that this would be of critical influence on their decision to use OS.

"Male pt F: “What other people think is not important. As long as I can walk without pain, then they can say whatever they like.”"

#### Interplay between factors of ‘usability’

Three factors were indicated as important for ‘usability’: improvement of walking, cosmetic appearance, and ease of use. Of these three factors, an improvement of walking was of most importance, and always had an influential role in a patient’s decision to use their OS. When an improvement of walking is achieved, patients felt they regained their freedom and independence.

"Female pt K: “You feel free, you can walk again. It’s such a relief.”"

An improvement of walking was described differently by patients, often depending on a patient’s pathology and their choice of wording. For example, they might highlight a reduction in pain, ease of walking, or comfort as being of most importance. However, when asked directly, patients clearly stated that these factors should not be viewed separately. Rather, the interplay of these factors together determines whether an improvement of walking can be achieved.

"Female pt A: “A good fit and less pain go hand in hand.”"

Two final factors had varied influence: cosmetic appearance was important for most (but not all) women and for half of the men; ease of use was rarely indicated as important, with most OS being easy to use. A compromise was seen in the interplay between these two factors and an improvement of walking.

"Female pt K: “But, all in all, the look is not important, how I walk is important.”"

"Female pt C: “Taking my shoes off is difficult, but it’s more important that the pain is gone.”"

#### Interplay between factors of ‘communication and service’

Five factors of ‘communication and service’ were important: i) taking the patient into account; ii) confidence in clinicians; iii) patient involvement; iv) speed of service; v) consistency in clinicians. There was little relevant interplay between these factors, all were considered as being important for good communication and service. However, large differences could be found in what style of communication was considered as being ‘good’. A communication style which is perfectly suited to one patient might be a completely wrong approach for another. This is exemplified with the following quotes of two female patients of a similar age:

"Female pt E: “Yes, really nice, and I felt very comfortable. I had to walk a little, then I could hear them talking about me, amongst themselves, all the different problems I had. I really liked it.”"

"Female pt I: “You basically just sit there and keep quiet, like you’re a child. I found it so patronising, to be there while they talked about me, but not to me.”"

#### Acceptance of OS

In addition to the factors and domains described, patients spontaneously indicated that they also had to accept their OS, the problems with their feet and their underlying condition. This acceptance had a major influence on their decision to use their OS.

"Female pt I: “I choose to look less attractive, but to have no pain. And after a few weeks, that finally clicked. You have to accept it in your mind first, then later in your heart.”"

"Male pt I: “You accept, after all, that it is made to how your feet are.”"

### Quantitative results

‘Usability’ was consistently ranked as most important, while ‘the opinion of others’ was of relatively limited importance (Table 2). Some patients indicated ‘communication and service’ to be equally important as ‘usability’, whereas others indicated that it was of no importance (Table 2). All but one patient reported that the aim of their OS (i.e. the functional goal why OS were prescribed) was the most the important factor influencing ‘usability’ (Table 3). Although goals were described differently (depending on pathology and choice of wording; Table 3), all patients essentially had the same underlying goal: an improvement of walking, to regain freedom and independence.

**Table 2:** The relative importance of the three domains

Domain	Mean ± SD	Range
Usability of OS	54 ± 15	34 – 100
Communication and service	35 ± 13	0 – 50
Opinion of others	12 ± 11	0 – 33

Note: scores can range from 0–100; the sum of the three domains is always 100.

*n* = 22, one participant is missing, because the disc was forgotten during the interview.

*OS* = custom-made orthopaedic shoes.

**Table 3:** Factor in the domain usability of custom-made orthopaedic shoes indicated by the participant to be most important

Factor	% (n)
Reduction of pain	30% (7)
Ease of walking	26% (6)
Increase in comfort	17% (4)
Reduction or prevention of wounds	9% (2)
More stability	9% (2)
Being able to do activities again	4% (1)
Easy donning and doffing	4% (1)

Note: even though described differently by participants, all factors in this table (except easy donning and doffing) refer to the main goal of the custom-made orthopaedic shoes: an improvement of walking.

### Triangulation

The findings of the qualitative data and the quantitative data were complementary. A patient’s decision to use their OS was influenced by various factors indicated as being important and by acceptance of their OS. Factors of ‘usability’ were more important than factors of ‘communication and service’; ‘the opinion of others’ was of limited importance. An improvement of walking was consistently indicated as most important factor of ‘usability’ in both the qualitative and the quantitative data. The importance of other factors (cosmetic appearance and ease of use) was determined by reaching a compromise between these factors and an improvement of walking.

## Discussion

A patient’s decision to use OS is influenced by the factor that is most important for an individual patient: an improvement of walking, to regain freedom and independence. This can, for example, be reached by a reduction in pain, prevention of ulceration, or by the comfort of OS. The interplay between these factors determines if an improvement of walking can be reached. In previous studies, other factors as cosmetic appearance and ease of use of OS have been found to be important as well [4,5,8,10,11]. However, patients did not have to choose between these factors and an improvement of walking in those studies. When forced to choose, it was found that patients were making compromises between these factors and an improvement of walking. The result of those compromises determines the importance of cosmetic appearance and ease of use, and thereby the influence of these factors on a patient’s decision to use OS.

In addition to factors of ‘usability’, it was found that a patient’s decision to use OS was influenced by acceptance of OS as well. This finding is in line with research concerning AT in general, where it has been stressed that patients who have accepted their disability are more likely to use their AT [15,19,20]. The main barrier in acceptance of OS was their visibility. According to patients, OS are the visible representation of their disability. It can be hypothesized that OS will be more easily accepted by patients who have accepted their disability. However, more research is needed to get a better grip on acceptance of OS, and to investigate the implications for clinical practice.

Communication and service of clinicians have been marked as pivotal moments, with the potential to influence a patient’s decision to use OS [9,10]. That is again shown in this study. When the relationship between the patient and the clinicians is seen as a partnership in order to achieve well-being, patients will feel taken into account and will have confidence in their clinicians [9,10]. This feeling may positively influence a patient’s decision to use OS. Communication is also the only way for a clinician to gain insight in the acceptance of a patient, and in the compromises the patient is willing to make with regard to cosmetic appearance and ease of use of OS. There is not one communication style that will ensure successful achievement of a partnership between the patient and the clinicians, as large individual differences in preferred style of communication in this study showed. An individual approach is therefore most important in clinical practice. If that is matched to the patient’s preferences, communication is the key for a clinician to positively influence a patient’s decision to use OS.

Since this is a study with a mixed-method design with priority on the qualitative part, the results should be regarded as inductive [17]. Part of the findings of the qualitative part could be triangulated with findings of the quantitative part, which confirmed the qualitative findings. However, more quantitative research is still necessary to quantitatively investigate all relations found.

Other limitations of this study are found in two causes of possible selection bias. First, patients were selected after they had been provided with OS. Patients who refused their OS straight-away were therefore not included in this study. This is a legitimate exclusion, as only reasons were investigated for a patient’s decision to use a pair of OS they already had been provided with. Second, due to privacy reasons, no insight was available in the number and characteristics of eligible patients to whom an information letter was sent. It is therefore not known if the included patients differ from the patients that were not willing to participate, which limits generalisation. However, the characteristics of the patients included in this study are comparable with another Dutch study in which a large and representative group of patients was included [5]. In our opinion, the issues raised in this study will be transferable to a larger group of patients.

**Figure 2:**
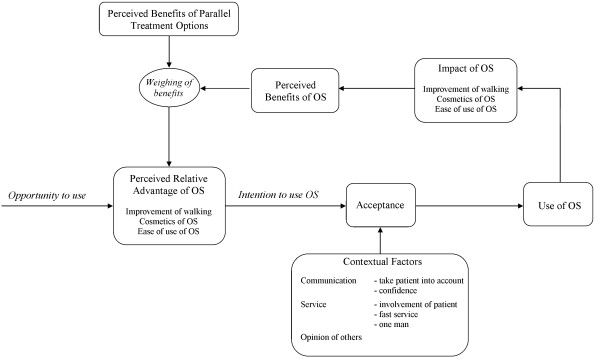
**Results applied to the conceptual model for assistive technologies outcomes research **[13]**.** Note: OS = custom-made orthopaedic shoes. Use of OS depends on the acceptance. Acceptance is influenced by factors of the perceived relative advantage and contextual factors that may be important for an individual patient. When OS are being used, the impact of OS determines the perceived benefits. These are weighed against the benefits of parallel treatment options, to determine again the perceived relative advantage.

The results of this study can be seen in a broader perspective, by applying them to a conceptual model for use of AT [13] (Figure 2). This shows that use of OS is influenced by acceptance of OS. Acceptance of OS is influenced by the perceived relative advantage (based upon factors of ‘usability’ important for an individual patient) and the contextual factors (‘communication and service’ and the ‘opinion of others’). When OS are being used, the impact of OS is determined by the outcomes of the factors of ‘usability’ of OS. This impact determines the benefits a patient perceives. The perception of these benefits is weighed against the potential benefits of parallel treatment options (if the patient has any options), to determine again the perceived relative advantage.

## Conclusions

A patient’s decision to use OS is influenced by those factors that patient indicates as being important and by acceptance of OS. An improvement of walking is the most important factor of ‘usability’, the importance of other factors of ‘usability’ (cosmetic appearance and ease of use) is determined by the compromises a patient makes between these factors and an improvement of walking. Communication is essential to gain insight in a patient’s acceptance and potential compromises, making communication the key for clinicians to influence a patient’s decision to use OS.

## Abbreviations

AT, Assistive technologies; OS, Custom-made orthopaedic shoes.

## Competing interests

The authors declare that they have no competing interests.

## Authors’ contributions

All authors participated in the design of the study. JvN carried out, transcribed, coded and analysed the interviews. KP coded seven interviews. JG analysed the interviews. All authors agreed on the theoretical framework and interpretation of the data. JvN drafted the manuscript, PD, JG, and KP helped to draft the manuscript. All authors read and approved the final manuscript.

## Pre-publication history

The pre-publication history for this paper can be accessed here:

http://www.biomedcentral.com/1471-2474/13/92/prepub
